# Randomized Clinical Trial: The Effect of Exercise of the Intrinsic Muscle on Foot Pronation

**DOI:** 10.3390/ijerph17134882

**Published:** 2020-07-07

**Authors:** Manuel Pabón-Carrasco, Aurora Castro-Méndez, Samuel Vilar-Palomo, Ana María Jiménez-Cebrián, Irene García-Paya, Inmaculada C. Palomo-Toucedo

**Affiliations:** 1 Cruz Roja, Nursing Department, University of Seville, 41009 Seville, Spain; mpabon@cruzroja.es; 2 Podiatry Department, University of Seville, 41009 Seville, Spain; ipalomo@us.es; 3 Virgen del Rocío Hospital, 41013 Seville, Spain; samuelvilarpalomo@hotmail.com; 4 Nursing and Podiatry Department, University of Malaga, 29071 Malaga, Spain; amjimenezc@uma.es (A.M.J.-C.); irenegpaya@uma.es (I.G.-P.); 5 Instituto de Investigación Biomédica de Málaga (IBIMA), 29010 Malaga, Spain

**Keywords:** foot posture index, pronation, short foot exercise, navicular drop

## Abstract

*Background:* There is little scientific evidence regarding the effectiveness of strengthening exercises on the foot’s intrinsic musculature in improving the lower limb on the statics and dynamics in healthy individuals. *Method:* To evaluate the effect on foot posture with regard to the reinforcement of the short foot exercise (SFE) compared to another without a recognized biomechanical action, which we called the “non-biomechanical function” (NBF) exercise. A randomized clinical trial was carried out with 85 asymptomatic participants with a bilateral Foot Posture Index (FPI) greater than 6 points. An experimental group (*n* = 42) did SFE training and a control group (*n* = 43) carried out NBF exercises. The foot posture was evaluated twice via the navicular drop (ND) test, and the FPI was assessed on the day of inclusion in the study (pre-intervention) and after four weeks of training (post-intervention). *Results:* Statistically significant values were not found in foot posture between the experimental and the control groups when comparing before and after the training. However, the foot posture was modified in both groups with respect to its initial state, and the ND value decreased. *Conclusions:* SFE could be considered a useful tool to deal with pathologies whose etiology includes excessive pronation of the foot.

## 1. Introduction

The intrinsic foot muscle (IFM) has a determinant role in the standing position and in walking. Its function is considered primordial in maintaining the plantar arch and controlling foot posture along with the rest of its anatomical structures (i.e., bones, ligaments, extrinsic muscles, and fascia) [1,2]. Weakness of the short or intrinsic muscle of the foot is related to greater incidence of pronation, fasciitis, sprains, and injuries of other body parts [3,4,5,6]. Therefore, its training is considered relevant to maintain the core system of the foot [7,8,9].

Some studies relate foot posture to different limb pathologies, such as chondromalacia patella [10]. Yet, the research shows contradictory results. There are studies that conclude that participants with a normal Foot Posture Index (FPI) score are more inclined to suffer pain in the patellar tendon with respect to subjects with a slight pronation [11]. However, other research correlates the foot’s pronated position with injuries due to overloading in the lower limb [10]. In contrast, others reveal a significant increase in the risk of suffering injuries due to overloading in the supinated foot as well as a greater technical skill in sports practice, as the increase of the rigidity of supinated feet helps the practice of activities such as indoor football [12].

On the other hand, although specific exercises have been developed to reinforce the foot’s intrinsic muscle [1], such as the reverse tandem gait, exercises with marbles, and flexion movements with a towel or a candle, the short foot exercise (SFE) is considered more effective in terms of postural balance and excessive pronation. SFE is a widely used training intervention that has been developed recently to improve ankle proprioception and global movement pattern, so as to elevate and support the medial longitudinal arch of the foot and to improve dynamic standing balance [1,3,13].

Daily practice of the SFE seems to improve the stability and the capability of absorbing shock and, thereby, optimizes the biomechanics of the lower limb [1,14,15,16]. In this line, a study evaluated an increase in the activity of hallux abducto valgus via electromyography, which is significantly greater during the SFE in comparison to the other traditional exercises. [17]

Different authors propose the strengthening of the IFM in the short term as part of treating lower extremity disorders. Pes planus alignment, plantar fasciitis, ankle instability, or patellar tendon pain may be successfully managed using this method. In athletes and runners, symptoms due to the overuse of distal structures may be prevented when medial longitudinal arch instability is detected [1,4,7,8,9,12,13,14]. Since the pronation of the foot is related to weakness of the intrinsic plantar muscles, by enhancing this musculature through exercises, it is believed that the associated hyper-pronation could be recovered or decreased [3].

Notwithstanding, there exists limited evidence to support these effects, and previous studies have reported contradictory results [4]. However, the SFE is considered a useful exercise for strengthening the IFM [17].

The aim of this research was to check if modifications in the foot’s pronation are produced after training for four weeks in a group of asymptomatic subjects with pronated foot posture. The dependent variable was assessed via the navicular drop (ND) test [18] and the FPI [19] before and after this period. This training enhanced the IFM daily through the SFE and was compared to another group of individuals of similar characteristics who carried out an exercise without a therapeutic indication for the IFM. The research hypothesis is that the SFE can modify the foot’s pronation after being implemented across a four-week period.

## 2. Methodology

### 2.1. Trial Design

This was a randomized clinical trial with a double blind technique. The participants were randomly assigned to an intervention group with the SFE or a control group that performed exercises without a recognized biomechanical action, named the non-biomechanical function (NBF) exercise.

The main researcher and the participants did not know to which group they had been assigned or which type of treatment would be undertaken in each group. The random assignment to each group was done by a collaborating researcher who established the sequence based on a one-by-one sequence generator (http://www.random.org).

### 2.2. Participants

**Figure 1:**
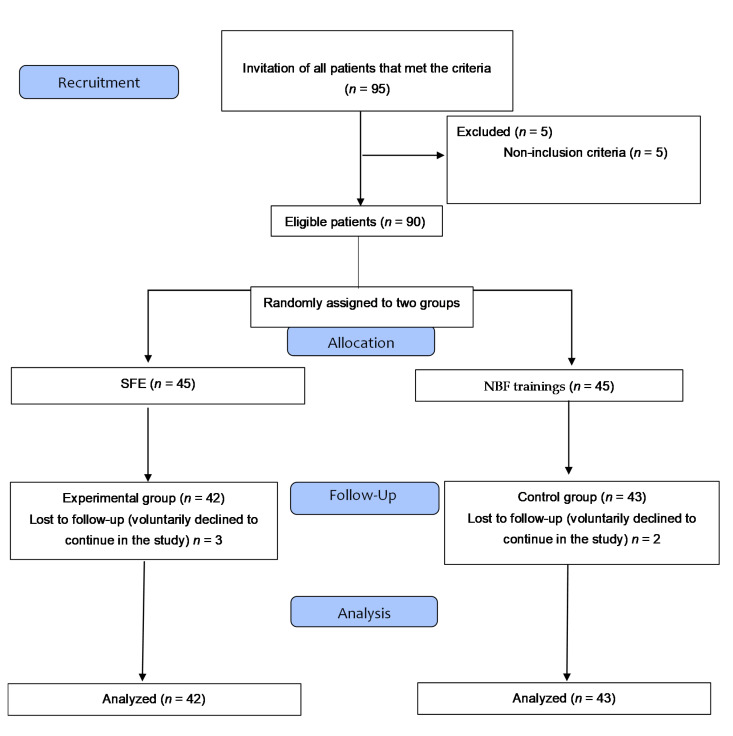
CONSORT flow diagram. SFE; short foot exercise. NBF; non-biomechanical function.

The participants were recruited from among university students, and the data were collected in the Clinical Area of Podiatry of the University of Seville and a private center of podiatry. The exclusion criteria were FPI less than 6 points in either the right or left feet (>6 points needed in both feet to identify pronator individuals) [19], any sign of pain in the lower limbs, current orthopedic treatment, pregnancy, ligamentous hyperlaxity, serious illnesses, osteoarticular surgery of the foot, and previous personal experience with the SFE. The participants (*n* = 90) who met the inclusion criteria and voluntarily expressed their wish to be part of the research (i.e., gave informed consent) were randomized into two study groups. Lastly, after removing the losses, a total of 85 subjects were divided into an experimental group (*n* = 42) that practiced IFM with the SFE, and a control group (*n* = 43) that performed active movements of the flexion and extension of the metatarsophalangeal joints of the foot without biomechanical action (Figure 1).

The Experimental Ethics Committee of the Seville University Hospital passed this research, which adhered to the Declaration of Helsinki [20], and it was registered according to the guideline of the Declaration CONSORT 2010 for randomized clinical trials [21]. The study was registered on Clinicaltrial.Gov with ID NCT03993470.

### 2.3. Interventions and Outcomes

The pronation of each foot of each participant (*n* = 180) was evaluated twice, pre-training (the day of the beginning the study) and post-training (after four weeks of muscle work). The ND test [18] and the FPI [19] were used as clinical valuation tools of the dependent variable.

To carry out the ND clinical valuation, a mark is initially made at the level of the navicular tuberosity (a ball-point pen dot in the skin). The subject is in an initial sitting position with his/her knees at 90°, and the distance from the tuberosity of the navicular bone to the ground is measured in millimeters. Then, the patient moves to a standing position with a relaxed load and the distance of the scaphoid tuberosity to the ground is again quantified. One measurement is subtracted from the other. In the cases in which this difference, expressed in millimeters, is ≥10 mm, the ND signifies an excessive pronation of the foot [18].

Foot Posture Index [19]:

The FPI quantifies the posture of each foot of the subject via a total of six items from a total score between −12 and +12 is obtained (reference values indicative of the foot’s posture: From 0 to +5 indicates a neutral position, from +6 to +9 indicates a pronated position, from +10 to +12 indicates a hyperpronated position, from −1 to −5 indicates a supinated position, and from −6 to −12 shows a highly supinated position). The six items that comprise the FPI are: (1) Palpation of the head of the talus, (2) supra curvature and lateral inframalleolar, (3) position of the calcaneus in the frontal plane, (4) prominence of the talo-navicular region, (5) congruence of the internal longitudinal arch, and (6) abduction or adduction of the forefoot with respect to the rearfoot.

The SFE and NBF trainings:

1. Exercise of the SFE Reinforcement—Experimental Group

**Figure 2:**
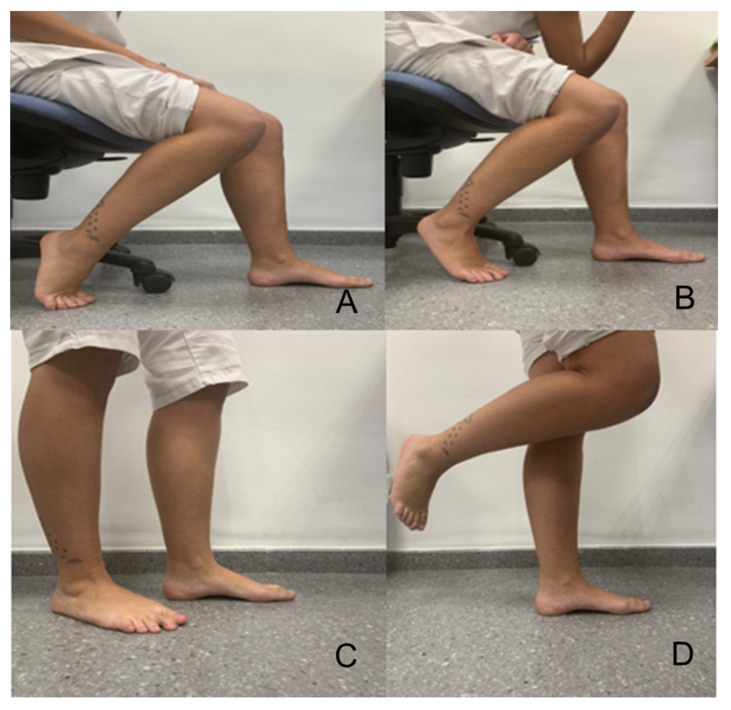
(**A**) Subject sitting—First week of training. (**B**) Subject sitting, support of the left elbow on the knee with the intention of loading on the foot (left) during training with the SFE—Second week of training. (**C**) Subject standing-position, training the left foot with the SFE—Third week of training. (**D**) Subject standing unipodal, with SFE training of the left foot—Fourth week of training. Unipodal standing-position. Seconds during the SFE, alternating with a period of rest at 10 s. A tot al of five series was carried out for each foot [17]. The researcher monitored and verified the daily exercise via telematic control (both groups were followed telematically).

In general, this training aims to bring the first metatarsophalangeal joint close to the calcaneus, keeping the toes extended (which is why it is called the short foot exercise) [17], and has to be done with one foot and then with the other. Daily practice was indicated in this study by carrying out the activity being modified from the 1st to the 4th week (a specific protocol of training, according to the literature, does not exist). Generally speaking, a 4-week to 8-week program is described according to the consulted authors [1,3,4,16,17]. The specific protocol used in this study was considered, according to our criteria. The most appropriate is outlined as follows. In the first week, the patient is in a seated position and performs flexion of the knee at 90°. During the second week, the subject is sitting and allowing loading until the foot performing the SFE moves away from the subject’s upper body. During the third week, the participant is standing. In addition, during the fourth week, the standing position is used on a single limb and, later, loading is on the other foot (Figure 2).

In all cases, training of the IFM was done daily, maintaining the position of maximum shortening of the foot for 30 s during the SFE, alternating with a period of rest of 10 s. A total of five series was carried out for each foot [17]. The researcher monitored and verified the daily exercise via telematic control (both groups were followed telematically).

2. NBF Control Group Exercise

The exercises indicated for the control group consisted of dorsal and plantar flexion of the metatarsophalangeal joints in unloading of the subject in a sitting position with the knee at 90° for a period of four weeks. The exercises were carried out daily without any kind of resistance (IE) for a period of 30 s while alternating with a period of rest of 10 s. A total of five series was completed for each foot. These exercises were considered as non-therapeutic, according to our knowledge, which might have affected the foot’s posture.

After the four-week training period, the dependent variables (i.e., FPI and ND) were evaluated and compared to the values obtained at the beginning of the study. Evaluation of the foot posture was conducted in both cases by a collaborating researcher with the intention of maintaining the main researcher’s blind status.

This study was carried out in accordance with the guidelines of the Declaration CONSORT 2010 for the notification of randomized clinical trials [21].

### 2.4. Statistical Analysis

The sample size was calculated for a power of 0.90, an alpha error of 0.05, and an effect size of 0.5 (test family: the G* Power 3.0.10, Franz Faul, Universidad Kiel, Germany) [22]. A total sample of 70 feet was estimated to be necessary for each group. This size was increased to compensate for any alterations in the statistical significance of the results caused by the possible abandonment of participants. A total of 90 subjects were initially recruited (i.e., 180 feet). By the end, five subjects had abandoned the research.

A previous descriptive analysis was performed with qualitative variables (i.e., the relative frequencies) and quantitative variables (i.e., measures of central tendency and dispersion). In turn, an exploratory analysis was carried out of all the data to identify the distribution of the variables (using the Shapiro–Wilk test), and a comparison between groups was made (i.e., a bivariate analysis of the qualitative variables with the chi-squared test, and of the quantitative variables via the Student’s *t-*test for independent groups after checking the normality). The Mann–Whitney U-test was applied when necessary, and the Wilcoxon test was used to assess the differences between paired samples (i.e., between groups). In the case of a comparison between two quantitative variables, the Pearson test was used for parametric samples and the Spearman test if the variable had non-parametric behavior [23].

All of the analyses were done using SPSS^®^ version 24.0. A *p*-value < −0.05 was established as statistically significant. An intention-to-treat analysis was done.

## 3. Results

*Description of the total sample and by groups*. The size of the final sample was *n* = 85 asymptomatic participants with pronated feet (a total of 170). The sample showed parity with respect to sex (53.3% women and 46.6% men). The average age was 20.26 ± 0.64 years and the body mass index (BMI) was 24.01 ± 0.34 (normal weight [24]). An analysis was conducted for the variables age, sex, BMI, ND, and FPI for each foot of the total sample both for the experimental and the control groups. The data are shown below (Table 1).

Table 1 shows a significant *p*-value in the baseline valuation with respect to the BMI and the FPI for the left foot.

The post-intervention analysis shows the descriptive results for the ND and FPI for the right and left feet of both groups after applying the follow-up period of the training plan for four weeks (the SFE for the experimental group and the NBF for the control group). Moreover, the general pre-intervention and post-intervention data were compared. These data are presented in Table 2.

Significant differences were noted when the data were evaluated without disaggregating the sample into the experimental and control groups upon which the differences were no longer significant.

Regarding the gender variable, no difference was found in any of the study variables before performing the exercises. However, in the control group, the post-intervention right FPI and ND values of women were significantly higher than those of men. We are not able to explain this finding since we have no previous literature regarding this topic, and the baseline of both groups and the gender proportions are similar.

The variable “differences of averages” was recoded. This enabled a comparison of the values of foot posture via the ND and FPI for the right and left feet between both groups with respect to the pre-intervention and post-intervention values. Analysis of the data did not show statistically significant differences for the two groups after applying the intervention in the experimental group with respect to the control group (ND: *p* = 0.124 and 0.392 for the right and left feet, respectively. FPI: *p* = 0.282 and 0.104 for the right and left feet, respectively). Table 3 shows the data. In no case is the value considered statistically significant (i.e., a *p*-value of <0.05).

Lastly, it was assessed whether or not there was an improvement in the control and experimental groups (Table 4).

An improvement in FPI was observed in both groups. Regarding the assessment of the ND, improvements only in the right foot were observed.

## 4. Discussion

The aim of this study was to evaluate if daily training of the short plantar muscle produced a significant modification in foot posture after four weeks of an SFE training plan in asymptomatic subjects with pronated foot posture when compared to a group of control subjects (i.e., training with NBF exercises).

Our results show that the SFE training did not produce a statistically significant difference in foot posture in comparison to the NBF exercise between the two groups. However, changes were observed in the ND and FPI pre-intervention and post-intervention for the subjects of each independent group. A tendency of a more neutral position and a decrease of the ND were noted.

The scientific literature argues the importance of IFM in the stabilization and maintenance of the core system of the foot, considering this to be a system of active, passive, and neurological integration of this structure [7,9,15,25]. The relevance of reinforcing the IFM in relation to the appearance of fasciitis, sprains, instability, or excessive pronation of the foot [7,26,27] and the influence of its strain on low values of the ND, which is indicative of excessive pronation [28], have been highlighted. This information is supported by studies such as that of Cheung et al. after examining 20 long-distance runners with and without plantar fasciitis, which indicates reduced muscle volume in the IFM of those experiencing pain compared to asymptomatic subjects [27].

In general, authors agree that IFM strengthening provides benefits related to the position, stability, and biomechanics of the foot. McKeon et al. suggested that such training should be included in rehabilitation programs for sport injuries [8,9]. In the same way, Sulowska et al. concluded that this is part of the training for runners and athletes in order to prevent overuse injuries [1,13].

Therefore, it is necessary to understand which type of exercise or protocol is more efficient and has better cost–benefit results. In this sense, diverse works have defended the SFE when compared to other types of training centered on improving the short plantar muscle [1,3,25,29,30] such as toe curl activity exercises [17], proprioceptive sensory exercises [26], and “Vele’s Forward Lean” and “Reverse Tandem Gait” [1]. Lee at al. argued that the SFE enabled good control of the foot’s pronation [29] and, in 2019 [26], defended this training (three times a week for a duration of eight weeks) as being more effective in preventing and recuperating from foot sprains when compared to proprioceptive exercises or even the use of plantar orthotics [15].

Gooding et al. observed that this specific training increased the volume and, therefore, the muscle activity by 16.7% ± 12.1% of the quadratus plantar muscle and by 34.9% ± 81% of the abductor muscle of the fifth toe, and, therefore, concluded that it produced better stabilization and maintenance of the core system of the foot after training with the SFE [31].

Following this argument, Moon et al. checked whether performing the SFE produced a tendency toward a more normalized posture of the foot [3]. This was similar to the work of Unver et al. who stated that enhancing the IFM with the SFE (for six weeks) tended to normalize the foot’s posture in flat feet with respect to the ND and the FPI [32]. These conclusions were similar to those obtained in other studies with shorter training periods (SFE for three minutes a day for four weeks) [9,33,34].

Many studies evaluated the muscle tone after performing the SFE and defended an increase in power of the muscles involved in maintaining the plantar arch [17,34,35].

As noted, there is no unified protocol that presents scientific evidence about how to train with the SFE (variations in the period of implementation of four to eight weeks, training daily, or three days per week [1,3,4,10,11,12,26,32]. Mignogna et al., after conducting a critical study in relation to three articles based on the SFE in healthy subjects, presented the limitations of the results obtained, which reached a scientific evidence level of 2. This led to them concluding that the SFE could not be defended as a training method, but that it could improve posture control. According to their conclusion, foot posture was influenced not only by the short plantar muscle, but by a set of anatomic structures (i.e., extrinsic muscle, ligaments, and articulations) [2,36].

Previous reference works mention situations of hyper-pronation of the foot, fasciitis, sprains, and other pathologies that cause pain and disability. We think that this factor has largely influenced the results. The inclusion criterion in this study was the absence of symptomology (i.e., asymptomatic students), which found, in our sample, that, after the analysis of foot posture, there were no significant results after training with the SFE. Martínez-Amat et al. drew similar conclusions after their research, upholding that, after sports training, no effect at all was seen on the footprint or index [37].

Recently, Namsawang et al. [38] investigated the effects of the SFE alone and after adding neuromuscular electrical stimulation in healthy people diagnosed with flexible flat feet. The navicular height using radiography and the cross-sectional area of the abductor hallucis (AbdH) muscle obtained by ultrasound machine and its activity with surface electromyography were evaluated. No significant differences were found in navicular height or the cross-sectional area of the AbdH between the control and experimental groups. However, the authors concluded that the SFE with neuromuscular electrical stimulation was more effective than the SFE alone in increasing AbdH muscle activity.

In summary, the arguments observed in the scientific literature support the SFE as a training method for reinforcing the IFM and, consequently, in controlling foot posture. However, these data have a greater significance in subjects with podiatric pathologies or symptoms.

As per the study of Okamura et al. [34] of patients with painless flexible flat feet, the results may be influenced by the characteristics of the sample. In our sample, participants were all asymptomatic and young despite having an FPI in pronation. On the other hand, the performance of continued exercises can contribute to an improvement of muscle tone among the participants in the control group. This may have caused a lack of differences between the groups. The researchers consider that other factors could have influenced the correct alignment of foot posture such as physical exercise and the footwear used. The SFE slightly but significantly corrected static foot alignment, and, thus, could prevent injuries related to pes planus alignment.

The development of a unified protocol in the carrying out of the SFE is considered necessary. It would also be interesting to evaluate the results in a sample of subjects who are older and who have previous pathologies as well as analyzing the results on static and dynamic foot and ankle kinematics.

Therefore, after this study, it is proposed to conduct works with subjects with pathologies caused by foot posture, or else with a sample of subjects who have hyper-pronated foot posture, to more significantly evaluate the tendency of foot posture compared to normal values.

## 5. Conclusions

The results of this research indicate that, in the sample of asymptomatic subjects who carried out the SFE, a statistically significant difference was not shown in foot pronation via the FPI and ND when compared to the control group, neither pre-intervention nor post-intervention. Nevertheless, a tendency to a more neutral index value of foot posture and a lower index of ND of all subjects in this study was noted. Therefore, the SFE could be considered a useful tool to deal with pathologies whose etiology includes an excessive pronation of the foot.

## Figures and Tables

**Table 1:** Initial values of the demographic variables of the whole sample and for both groups as well as the navicular drop (ND) and Foot Posture Index (FPI) variables for right and left feet (average ± standard deviation).

Sample *N* = 85		Group		
		Experimental *n* = 42	Control *n* = 43	*p-*Value
Gender Female	48 (53.3%)	18 (57.1%)	25 (42.8%)	
Male	42 (46.6%)	24 (57.1%)	18 (42.8%)	2.00, *p* = 0.156 ^b^
Age	20.26 ± 0.64	19.45 ± 0.38	20.92 ± 1.1	1.64, *p* = 0.110 ^a^
BMI	24.01 ± 0.34	24.13 ± 4.16	21.65 ± 3.35	722.0, *p*= 0.032 ^c^
ND, right foot	0.72 ± 0.05	0.79 ± 0.08	0.67 ± 0.06	−1.97, *p* = 0.057 ^a^
ND, left foot	0.65 ± 0.05	0.70 ± 0.06	0.65 ± 0.07	803.0, *p* = 0.078 ^c^
FPI, right foot	6.57 ± 0.41	6.77 ± 0.62	6.35 ± 0.31	−1.74, *p* = 0.085 ^a^
FPI, left foot	6.68 ± 0.28	6.94 ± 0.52	6.27 ± 0.22	−3.30, *p* = 0.050 ^a^

^a^ Student’s *t*-test. ^b^ Chi-squared test. ^c^ Mann–Whitney U-test. Values are presented as mean ± standard deviation (SD). BMI: body mass index.

**Table 2:** Descriptive statistical analysis after the training period of four weeks for the total sample and both groups.

Sample *N* = 85 *p*-Value			Group		
			Experimental *n* = 42	Control *n* = 43	*p*-Value
ND, right foot	0.61 ± 0.04	*p* = 0.001 ^b,^*** r = 0.700 ^c^	0.63 ± 0.06	0.59 ± 0.54	−1.20 *p* = 0.403 ^a^
ND, left foot	0.56 ± 0.15	*p* = 0.037 ^e,^* r = 1.00 ^c^	0.49 ± 0.32	0.59 ± 0.06	818.00 *p* = 0.240 ^d^
FPI, right foot	5.44 ± 0.37	*p* = 0.001 ^b,^*** r = 0.884 ^c^	5.37 ± 0.63	5.43 ± 0.44	−0.985 *p* = 0.495 ^a^
FPI, left foot	5.15 ± 0.37	*p* = 0.001 ^b,^*** r = 0.800 ^c^	5.09 ± 0.66	5.19 ± 0.42	−1.57 *p* = 0.276 ^a^

^a^ Student’s *t*-test. ^b^ Pearson test. ^c^ r = correlation index. ^d^ Mann–Whitney U-test. ^e^ Spearman test. Significance set at *p* < 0.05: * *p* < 0.05, *** *p* < 0.001.

**Table 3:** Statistical analysis of the pre-intervention and post-intervention Navicular Drop and Foot Posture Index (FPI) values of the right and left feet of the samples and both groups.

Sample *N* = 85		Group		
		Experimental *n* = 42	Control *n* = 43	*t o U /p*-Value
Differences in ND, right foot	−0.11 ± 0.03	−0.16 ± 0.06	−0.04 ± 0.04	−1.85 *p* = 0.124 ^a^
Differences in ND, left foot	−0.09 ± 0.14	−0.21 ± 0.31	−0.03 ± 0.06	832.000 *p* = 0.392 ^b^
Differences in FPI, right foot	−1.13 ± 0.21	−1.40 ± 0.31	−0.92 ± 0.30	−1.30 *p* = 0.282 ^a^
Differences in FPI, left foot	−1.48 ± 0.52	−1.85 ± 0.37	−1.08 ± 0.28	−1.50 *p* = 0.104 ^a^

^a^ Student’s *t*-test. ^b^ Mann–Whitney U-test.

**Table 4:** Assessment of the FPI and ND within the same study groups.

	Experimental	Effect Size	Control	Effect Size
ND Right_POST–ND Right _PRE	0.000 ^a^	0.56 ^c^	0.000 ^a^	0.25 ^c^
ND left_POST–ND left _PRE	0.131 ^b^		0.227 ^b^	
FPI Right_POST–FPI Right _PRE	0.000 ^a^	1.02 ^c^	0.000 ^a^	0.39 ^c^
FPI left_POST– FPI left _PRE	0.000 ^a^	1.12 ^c^	0.000 ^a^	0.77 ^c^

^a^ Student’s *t*-test. ^b^ Wilcoxon test. ^c^ Cohen’s D.
